# Supplementary material for: Complexome profiling of the Chlamydomonas psb28 mutant reveals TEF5 as an early PSII assembly factor
Source: Plant Cell. 2025 Mar 17;37(6):koaf055. doi: 10.1093/plcell/koaf055 (PMC12164590; doi:10.1093/plcell/koaf055)
Supplement: koaf055_Supplementary_Data [file koaf055_supplementary_data.zip › psb28_interactive.zip!/plots/intensity/qualityPlots/peptides/TIM44_Cre16.g660800.t1.2.html]

### Cre16.g660800.t1.2

Trivial name: TIM44  
SeqCoverage [median]: 32.20  
Mass: 44.23 kDa  
  
 

| Sequence | MissedCleavage | Mass [Da] | wtIntensity | muIntensity | Score |
| --- | --- | --- | --- | --- | --- |
| AGAAAGAAAGAAGGAGAGAGGEPAAQR | 0 | 2035.98 | 4643.40 | 9167.41 | 134.66 |
| AGFEDLK | 0 | 778.39 | 0.00 | 704.94 | 85.27 |
| AYTGPVAAATYDGPTALVLAK | 0 | 2049.08 | 1171.27 | 0.00 | 85.51 |
| DKFGNVVDGDANTIQR | 1 | 1747.85 | 2149.84 | 5857.14 | 122.13 |
| ETLLPAQDITSATR | 0 | 1514.79 | 2895.35 | 14042.40 | 125.22 |
| FGNVVDGDANTIQR | 0 | 1504.73 | 0.00 | 1247.28 | 127.87 |
| FVQSVK | 0 | 706.40 | 0.00 | 2005.77 | 94.41 |
| LIQTLAQDLR | 0 | 1169.68 | 530.29 | 1736.94 | 92.44 |
| SPVVTAEGK | 0 | 886.48 | 4478.03 | 10319.15 | 149.82 |
| SSMFTGSEASR | 0 | 1158.50 | 0.00 | 536.89 | 68.89 |
| VRDQAFDMNR | 1 | 1250.58 | 2030.83 | 3139.88 | 71.07 |
|  |  |  | 17899.0 | 48757.8 |  |
